# Capsaicin-Induced Activation of p53-SMAR1 Auto-Regulatory Loop Down-Regulates VEGF in Non-Small Cell Lung Cancer to Restrain Angiogenesis

**DOI:** 10.1371/journal.pone.0099743

**Published:** 2014-06-13

**Authors:** Samik Chakraborty, Arghya Adhikary, Minakshi Mazumdar, Shravanti Mukherjee, Pushpak Bhattacharjee, Deblina Guha, Tathagata Choudhuri, Samit Chattopadhyay, Gaurisankar Sa, Aparna Sen, Tanya Das

**Affiliations:** 1 Division of Molecular Medicine, Bose Institute, Kolkata, West Bengal, India; 2 Department of Biotechnology, Viswa-Bharati, Santiniketan, West Bengal, India; 3 National Center for Cell Science, Pune University, Pune, Maharashtra, India; 4 Department of Microbiology, Lady Brabourne College, Kolkata, West Bengal, India; Winship Cancer Institute of Emory University, United States of America

## Abstract

Lung cancer is the leading cause of cancer-related deaths worldwide. Despite decades of research, the treatment options for lung cancer patients remain inadequate, either to offer a cure or even a substantial survival advantage owing to its intrinsic resistance to chemotherapy. Our results propose the effectiveness of capsaicin in down-regulating VEGF expression in non-small cell lung carcinoma (NSCLC) cells in hypoxic environment. Capsaicin-treatment re-activated p53-SMAR1 positive feed-back loop in these cells to persuade p53-mediated HIF-1α degradation and SMAR1-induced repression of Cox-2 expression that restrained HIF-1α nuclear localization. Such signal-modulations consequently down regulated VEGF expression to thwart endothelial cell migration and network formation, pre-requisites of angiogenesis in tumor micro-environment. The above results advocate the candidature of capsaicin in exclusively targeting angiogenesis by down-regulating VEGF in tumor cells to achieve more efficient and cogent therapy of resistant NSCLC.

## Introduction

Highly resistant non small-cell lung carcinoma (NSCLC) that comprises 80% of all lung cancers is intrinsically resistant to chemotherapy and/or irradiation therapy. Since, angiogenesis is essential for NSCLC growth and metastasis, therefore controlling tumor-associated angiogenesis can be a promising tactic in limiting NSCLC progression. Several pro-angiogenic factors such as vascular endothelial growth factor (VEGF) are highly expressed in the tumor microenvironment and strongly induce tumor angiogenesishttp://www.ncbi.nlm.nih.gov/pmc/articles/PMC3827603/ - b3 [1]. This shift of the tumor microenvironment to an angiogenic state, or “angiogenic switch” [1], [2], is an important rate limiting factor in tumor development.

Expression of the VEGF gene has been shown to be upregulated by hypoxia [3]–[5] and turnover of VEGF is mediated by the hypoxia-inducible factor-1 (HIF-1) [2], [3]. Under normoxic conditions, HIF-1α levels are strongly regulated by oxygen tension through hydroxylation of prolyl residues, while hypoxic conditions hinder prolyl hydroxylation of HIF-1α [4] and the protein is stabilized, enabling it to transactivate target genes like VEGF [3]. A wealth of reports tightly link HIF-1α to p53 in an inverse relationship in which p53 inhibits HIF-1α transcription [6] and induces its degradation under several sub-cellular conditions of stress [7] thereby http://www.jbc.org/search?author1=Joanna+Zawacka-Pankau&sortspec=date&submit=Submitresulting in its potent repression. Interestingly, p53 is stabilized by SMAR1, a scaffold matrix-associated region-binding protein, through displacement of Mdm2 from p53 N-terminal pocket and hence rescuing p53 from the Mdm2-mediated proteasomal degradation [8]. Contemporary reports [9], [10] demonstrate that on mild DNA damage SMAR1 promotes p53 deacetylation through recruitment of HDAC1 and specifically represses Bax and Puma expression thereby inhibiting apoptosis. These reports not only attest the candidature of SMAR1 in modulating the activity of p53 but also raise the possibility of involvement of p53 in other cellular functions in the mild DNA-damaging micro-environment of the cell. Importantly, several studies have also identified complex cross-talks between p5*3* and Cox-2, whereby Cox-2 suppresses p53-network in cancer cells [11], [12] and *vice versa* [12]. All these information tempted us to render insight into the existence of an interactive relationship between SMAR1, p53, Cox-2 and HIF1-α, if any, that decides fate of VEGF expression during tumor angiogenesis in NSCLC. Inhibition of tumor growth by anti-angiogenic agents has been achieved where promising antitumor responses have been reported for a variety of anti-VEGF agents. However, toxicity of most of these drugs as well as development of resistance towards those agents signify the necessity of identifying alternative non-toxic agents to achieve the continuous inhibition of angiogenesis for effective NSCLC therapy.

Increasing evidence indicated the anticancer effects of capsaicin (8-methyl-*N*-vanillyl-6-nonenamide), the active component of chili pepper, against various cancers [13]–[18]. Capsaicin has been reported to provoke apoptosis and restricts benzo(a)pyrene induced lung tumorigenesis in Swiss albino mice [19] and alleviates the imbalance in xenobiotic metabolizing enzymes and tumor markers [20]. According to Anandakumar *et al.* [20] capsaicin modulates pulmonary antioxidant defense system during benzo(a)pyrene-induced lung cancer in Swiss albino mice [19]. Furthermore, capsaicin displays anti-proliferative activity against human small cell lung cancer in cell culture and nude mice models via the E2F pathway [21]. Recent report from our laboratory has shown the apoptogenic effect of capsaicin on human NSCLC cells [22]. However, there is no report on the role of this phytochemical on NSCLC-induced angiogenesis. In addition, although capsacin has been shown to suppress human fibrosarcoma-induced angiogenesis in chick chorioallantoic membrane assay [23] by inhibiting VEGF-induced proliferation, and capillary-like tube formation of primary cultured human endothelial cells, the effect of this phytochemical on the VEGF expression in NSCLC cell has not yet been explored in detail.

Our results signify that while impediment of p53-SMAR1 loop induced VEGF expression in NLCSC cells thereby favoring endothelial cell (EC) migration and network formation in tumor environment, reframing of these pro-angiogenic signals by capsaicin blocked VEGF production even under hypoxic condition, thereby restraining NSCLC-induced net work formation by the endothelial cells. Such development in understanding may offer the panorama of exclusively targeting pro-angiogenic factors and pathways to achieve more efficient and cogent lung cancer therapy.

## Materials and Methods

### Cell culture

Human cancer cell lines, A549, MCF-7, HeLa, HCT-15, and normal lung fibroblast WI-38, were obtained from National Centre for Cell Science, India, and maintained at 37°C and 5% CO_2_ in DMEM medium supplemented with 10% FBS (Lonza, NH), L-glutamine (2 mM), sodium pyruvate (100 µg/ml), nonessential amino acids (100 µM), streptomycin (100 µg/ml), penicillin (50 U/ml; Invitogen, CA) [24]. Human umbilical vein endothelial cells (HUVEC) were obtained from HIMEDIA Cell Culture (Mumbai, India), and maintained at 37°C and 5% CO_2_ in M199 (Gibco, Grand Island, NY) supplemented with 20% FBS and EC growth supplement (ECGS; BD Biosciences, Bedford, MA). All experiments were performed with HUVECs between passages 2–5. Viable cell numbers were determined by Trypan blue exclusion test [11].

To mimic hypoxia, A549 cells were grown in presence of 100 µmol/L CoCl_2_ (Sigma, St Louis). Cells were treated with capsaicin (Sigma, St. Louis) as required in experiment. When required, A549 cells were treated with Brefeldin A (1 µg/ml, Calbiochem, NJ) and/or MG 132 (10 µM, Sigma, St Louis) and/or recombinant VEGF (0.8 ng/ml, to keep equivalent amount of VEGF that was present in Hy-A549 spent media which was used at 1∶1 ratio with M199 on endothelial cell; Peprotech, CA) and/or VEGF neutralizing antibody (25 ng/ml, R&D Biosystems, MN), 6 hours before harvesting cells.

### Wound healing assay

Wound healing assay was performed to assess the migration of HUVECs. Cells were seeded in 12-well plates and allowed to form confluent monolayers. The monolayers were then scratched horizontally using a sterile 100 µl pipette tip. Then cells were cultivated in medium containing 0.5% FBS overnight and exposed to different concentration of agents. Cells were washed with PBS and cultivated in DMEM medium. Bright field images of the randomly selected views along the scraped line were taken. Migration was quantified by a semi-automated computer-assisted procedure by a person blinded with respect to the experimental-treatment. The data from triplicate wells were calculated as the mean +/− SEM. The migration rate of control cells was taken as 100% and that of other plates were compared with control cells [25].

### Sprout formation assay

Matrigel Matrix (BD Biosciences, Heidelberg, Germany) were thawed at 4°C overnight. 12-well plate was coated with 500 µl Matrigel/per well and incubated at 37°C for 20 min for polymerization. HUVECs (1×10^5^) were re-suspended in 500 µl M199 containing 2% FBS and different concentration of agents, and seeded onto the Matrigel-coated plate. After 6–8 h incubation, HUVECs in control group formed tube-like structure, which were defined as endothelial cord formations that connected at both ends. Tube formation was visualized by microscopically (Olympus IX71) and photographs of 10 randomly selected fields were captured by CCD camera.

### Western blotting and co-immunoprecipitation

Cells were homogenized in Hepes buffer (20 mM Hepes, pH 7.5, 10 mM KCl, 1.5 mM MgCl_2_, 1 mM Na-EDTA, 1 mM Na-EGTA, and 1 mM DTT) supplemented with protease and phosphatase inhibitor mixtures [24]. Total protein concentration of cell lysate was estimated by Lowry's method. Equal amounts of protein were subjected to SDS-PAGE, and then electrically transferred onto PVDF membranes (Millipore, Darmstadt, Germany). Subsequently, the membrane was blocked for 1 h with 5% non-fat milk in TBS-0.1% Tween 20 (TBST) and probed with specific antibodies like, anti-VEGF, anti-TGF-β, anti-EGF, anti bFGF, anti-HIF-1α, anti-p53, anti-Cox-2,anti-SMAR1/BANP, anti-H1-Histone and anti-α-Actin (Santa Cruz, CA), anti-phospho-ser15-p53, anti-EGFR (Cell signalling, MA). The protein of interest was visualized by chemiluminescence (GE Biosciences, NJ) [24]. For co-immunoprecipitation, immunocomplexes from whole cell lysate were purified using specific antibody and protein A-Sepharose beads (Invitrogen, MD) and immunoblotted. The protein of interest was visualized by chemi-luminescence. Equivalent protein loading was verified using anti-α-actin antibodies.

### Flow cytometry

For determination of apoptotic cell death, cells were stained with propidium iodide (PI) and Annexin-V-FITC (BD Pharmingen, CA) and analyzed on flow cytometer (FACS Callibur, BD Biosciences, CA). Electronic compensation of the instrument was done to exclude overlapping of the emission spectra. Total 10,000 events were acquired for analysis using CellQuest software (BD Biosciences, CA) [26]. Annexin-V-positive cells were regarded as early apoptotic cells [24]. For quantification of intracellular VEGF, cells pre-treated with Brefelidin A were harvested, washed with PBS, fixed with 4% para-formaldehyde for 10 min and permeabilized with 0.1% Triton-X-100 for 5 min and incubated with anti-VEGF antibody followed by TRITC-conjugated secondary antibody. Fluorescence was determined flow cytometrically using CellQuest software (Becton Dickinson, San Jose, CA).

### Fluorescence imaging

Cells grown on a cover slip were fixed with 4% para-formaldehyde and were stained with specific antibody after permeabilization with Triton X100, followed by TRITC/FITC-conjugated secondary antibody and visualized with confocal microscope (Carl Zeiss, Germany).

### ELISA

The conditioned medium of the cells was collected and clarified by centrifugation at 2,000 rpm for 10 min. Concentration of the protein of interest was measured using the ELISA kit according to the manufacturer's instruction (Ray Biotech, GA).

### RT-PCR assay

Following Saha *et al*. [11], 2 µg of the total RNAs extracted with Trizol reagent (Invitrogen, CA, USA) was reverse transcribed and subjected to PCR using RTplus PCR system (Eppendorf, Hamburg, Germany) an GeneAmp PCR system 2720 (Applied Biosystems; Foster city, CA). The resulting cDNAs were amplified with primers specific for VEGF 5′-GAGATGAGCTTCCTACAGCAC-3′(forward) and 5′-TCACCGCCTCGGCTTGTCACAT-3′(reverse), p53 5′-CCCACTCACCGTACTAA-3′ (forward) and 5′-GTGGTTTCAAGGCCAGATGT-3′ (reverse), HIF-1α 5′-TGGTGACATGATTTACATTTCTGA-3′ (forward) and 5′-AAGGCCATTTCTGTGTGTAAGC-3′ (reverse), SMAR1, 5′-GCATTGAGGCCAAGCTGAA-AGCTC-3′ (forward) and 5′-GGAGTTCAGGGTGATGAGTGTGA C-3′(reverse), Cox-2 5′-TGAT-CGAAGACTACGTGCAACA-3′ (forward) and 5′-GCGGATGCCAGTGATAGAGTG-3′ (reverse) and GAPDH (internal standard) 5′-CAGAACATCATCCCTGC-CTCT-3′ (forward), 5′-GCTT-GACAAAGTGGTCGTTGA-G-3′ (reverse).

### Plasmids and siRNA transfections

pcDNA3.1 p53, pcDNA3.1 SMAR1 and pcDNA3.0 Cox-2 or SMAR1-shRNA (300 pmole/million cells),and control pcDNA3.0 vectors (2 µg/million cells) were introduced into exponentially growing cancer cells using lipofectamine-2000 (Invitrogen, CA) according to the protocol provided by the manufacturer. Stably expressing clones were isolated by limiting dilution by selection with G418 (400 µg/ml; Cellgro, USA) and puromycin (1 µg/ml; Cellgro, USA) for 14 days, and cells surviving this treatment were cloned and assessed for p53, SMAR1 and Cox-2 by immunoblotting. For endogenous silencing of specific genes, cells were transfected with 300 pmol of HIF-1α-/Cox-2 -siRNA (Santa Cruz, CA) and p53 shRNA (Santa Cruz, CA) using lipofectamine-2000 for 12 h. The mRNA and protein levels were determined by RT-PCR and western blotting.

### Chromatin Immunoprecipitation and PCR

The ChIP assay was performed as previously reported by our laboratory [9]. Briefly, agarose beads were blocked with BSA and, following washing, the beads were pre-incubated with antibody against SMAR1/BANP (BTG-3 associated nuclear protein; Santa Cruz, CA). The cell lysates were sonicated to shear the DNA to lengths between 200 and 1000 base pairs and then centrifuged at 13,000 rpm for 10 min at 4°C. Supernatants were diluted 10-fold in ChIP dilution buffer and added to the pelleted agarose beads that were pre-incubated with antibodies. Following overnight incubation at 4°C, the beads were washed with low salt, high salt, LiCl and Tris/EDTA buffers. Finally, the chromatin was eluted by incubating the beads with 5 M NaCl at 65°C and proteins were removed by treatment with proteinase K. ChIP DNA was then purified using an appropriate purification kit and stored at −20°C. SMAR1-linked ChIP DNA was amplified using PCR. The sequences of probable SMAR1 binding sites on Cox-2 promoter are as follows: site-1: 5′-TGA-CCAGCATCCCAAATGTA-3′ (forward) and 5′-TGAGGGA-AAAACAGGGCATA-3′ (reverse); site-2 5′-CAAAAAGAAAATGA-TCCACGC-3′ (forward) and 5′-TCATGAGACACGGATGCCTA-3′ (reverse); site-3 5′-CCGTGTCTCA-TGAGGAATCA-3′ (forward) and 5′-ATCATGGGTAGTGCTCAGGG-3′ (reverse); site-4 5′-TGCT-GTCATTTTCCTGAATGC-3′ (forward) and 5′-GGGGATTTTGACAGTTGGAA-3′ (reverse); site-5 5′-GCCCAGGCA-ACTGAAAAGTA-3′ (forward) and 5′-CTCCCTGATGCGTGGATTAT-3′ (reverse); site-6 5′-TTT-TGGACATTTAGCG-TCCC-3′ (forward) and 5′- TGTTCTCCGTACCTTCA -CCC-3′ (reverse); site-7 5′-TACCTTTCCC-GCCTCTCTTT-3′ (forward) and 5′-TGGGGCGAGTA-AGGTTAAGA-3′ (reverse); site-8 5′-AAC-CTTACTCGCCCCAGTCT-3′ (forward) and 5′- CAGA-AGGACACTTGG-CTTCC-3′ (reverse).

### Quantitative real-time PCR

Quantitative real-time PCR was done to quantify the time dependent expression levels of VEGF and HIF-1α in capsaicin-treated Hy-A549 cells (22). Quantitative real time PCR was performed in Master cycler gradient (an Applied Biosystems 7500 Sequence Detection System) using SYBR-green Rox mix (ABgene, Epsom, United Kingdom). Primers used for VEGF are 5′-TCACAGGTACAGGGATGAGGACAC-3′ (forward) and 5′-CAAAGCACAG-CAATGTCCTGAAG-3′(reverse) and for HIF-1α 5′-TGGTGACATGATTTACATTTCTGA-3′ (forward) and 5′-AAGGCCATTTCTGTGTGTAAGC-3′ (reverse).

### Statistical analyses

Values have been shown as standard error of mean (SEM) or representative of typical experiment except otherwise indicated. Data were analyzed, and when appropriate, significance of the differences between mean values was determined by Student's *t* test. Results were considered significant at *p*<0.05. All experiments were performed independently three times.

## Results

### Capsaicin inhibits NSCLC-induced EC migration and network formation

**Figure 1:**
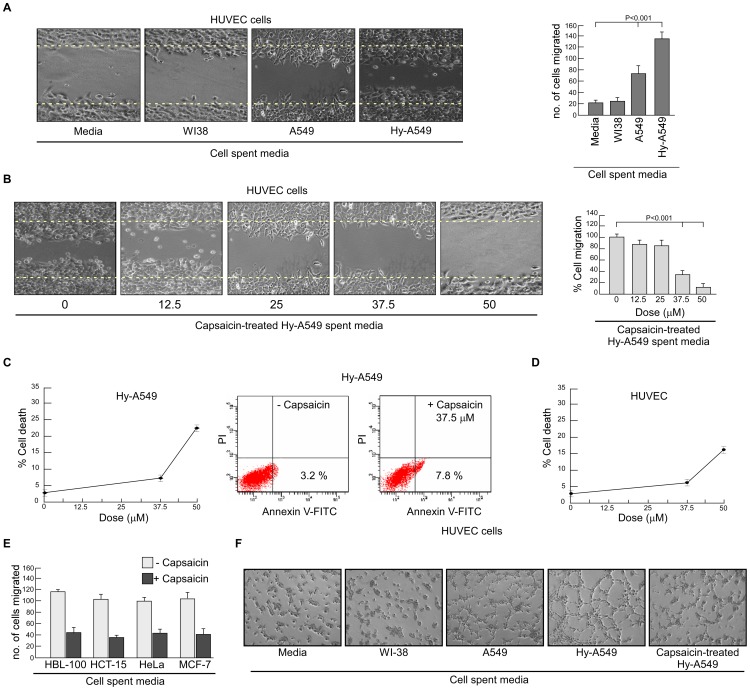
Effect of capsaicin on lung cancer cell spent medium-induced endothelial cell migration and network formation. (A) Migration of HUVECs in presence or absence of spent media of NME, WI-38, A549 and Hy-A549 (CoCl_2_-stimulated to mimic hypoxic condition required for tumor-induced angiogenesis) were subjected to bi-directional wound healing assay for 24 h (*left panel*). The number of cells migrated in the wound area are represented graphically (*right panel*). (B) Representative images of HUVEC migration upon incubation with capsaicin-treated (0–50 µM) Hy-A549 cell spent medium (*left panel*). Percent cell migrated in the wound area has been represented graphically (*right panel*). (C) Hy-A549 cells were treated with capsaicin in a dose-dependent manner for 24 h and cell viability was scored by trypan blue dye-exclusion assay (*left panel*). Hy-A549 cells, treated with capsaicin (37.5 µM), were subjected to Annexin-V-FITC/PI binding and analyzed flow cytometrically for the determination of percent early apoptosis (*right panel*). (D) Cytotoxic effect of different doses of capsaisin on HUVEC cells were measured by trypan blue dye-exclusion assay. (E) Graphical representation of HUVEC migration upon incubation with spent media from capsaicin-treated (37.5 µM) HBL-100, HCT-15, HeLa and A549. (F) Representative images of capillary-like sprout formation by HUVECs in presence of media alone or spent media of WI-38/A549/Hy-A549/capsaicin-treated Hy-A549. Values are mean ± SEM of three independent experiments in each case or representative of typical experiment.

Since EC migration is a pivotal step for tumor-induced angiogenesis; we investigated the migration of HUVECs in the presence of the spent media from cultures of both normal cells and NSCLC. In gist, HUVECs were cultured in the absence or presence of spent media of normal lung fibroblast cells (WI-38) and NSCLC cells (A549) as well as CoCl_2_-stimulated A549 cells (Hy-A549). Results of wound healing assay exhibited non-significant migration of HUVECs in the presence of spent media of WI-38 cells, whereas, significant migration was observed in the presence of A549 spent media, a situation mimicking the tumor-bearing condition (Figure 1A). Percent migration of HUVECs was even more in Hy-A549 spent media (Figure 1A) indicating that the hypoxic condition favors angiogenesis. Hy-A549 cells were, therefore, used for rest of the experiments to specify hypoxic condition.

Interestingly, spent-media of capsaicin-treated Hy-A549 significantly inhibited tumor-induced HUVEC migration in a capsaicin dose-dependent manner, the optimum effect being at 37.5 µM of capsaicin at 24 h beyond which no further significant change could be obtained (Figure 1B). The effect of capsaicin on the viability of Hy-A549 cells was examined by trypan blue dye-exclusion assay. Figure 1C (*left panel)* depicted the dose-dependent effect of capsaicin on the viability of Hy-A549 cells as well as HUVEC (Figure 1D). Results indicated that on exposure for 24 h, capsaicin did not induce any significant death in either of the cells till concentrations of 37.5 µM. These results thereby ruled out the possibility of retardation of EC migration due to Hy-A549 cell death at this concentration of capsaicin. However, concentrations beyond 37.5 µM were found to be toxic for both Hy-A549 cells and HUVEC (Figure 1C, *left panel* and 1D). Further experiments were therefore, restricted to 37.5 µM. To assess whether the reduction in cell number is due to early apoptosis, the number of Annexin-V positive cells was determined by flow cytometry. Results of Figure 1C (*right panel*) demonstrated 37.5 µM dose of capsaicin as sub-apoptotic dose for Hy-A549 cells. These results together ensured the absence of ‘interference’ of apoptotic cells at 37.5 µM dose of capsaicin used in the subsequent experiments. That the effect of capsaicin is not restricted to a specific type, was validated using a battery of cell lines, i.e., HBL-100, HCT-15, HeLa and, MCF-7 (Figure 1E). However, for detailed mechanistic studies experiments were performed with Hy-A549 cells.

Next, the sprout formation assay of ECs was performed on Matrigel, a well-established angiogenesis assay. HUVECs formed tube-like networks within 8 h (Figure 1F). Angiogenic sprout formation of HUVECs was highest in the presence of spent media of Hy-A549 cells followed by that of A549 cells and WI-38 cells (Figure 1F). However, capsaicin pre-treatment of Hy-A549 effectively hindered sprout formation by HUVECs (Figure 1F) where tube-like structures were reduced both in width and in length and showed incomplete as well as broken network structures (Figure 1F).

### Capsaicin inhibits VEGF to retard cancer cell-induced EC migration

**Figure 2:**
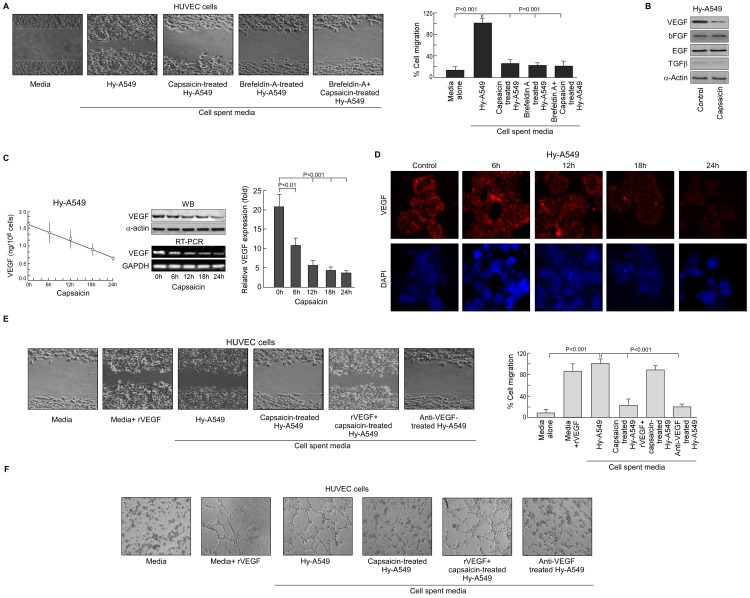
Capsaicin selectively inhibits VEGF secretion to retard Hy-A549 cell-induced HUVEC cell migration. (A) Representative phase contrast photomicrographs demonstrating HUVEC migration upon incubation with spent media of Brefeldin-A-pretreated, capsaicin (37.5 µM)-treated Hy-A549 cells (*left panel*). Percent cell migrated in the wound area has been represented graphically (*right panel*). (B) Immunoblots showing expression profiles of pro-angiogenic factors VEGF, bFGF, EGF, TGF-β, in presence or absence of capsaicin. (C) Secreted VEGF from cell-free supernatant of Hy-A549 was quantified by ELISA assay (*left panel*). Time-dependent expression profiles of VEGF-mRNA/-protein in capsaicin-treated Hy-A549 cells were determined by Western blot and RT-PCR respectively (*middle panel*). Capsaicin-treated Hy-A549 cells were examined for time-dependent variation in the expression profiles of VEGF by quantitative real time PCR analysis and represented graphically (*right panel*). (D) Immuno-fluorescent images (60x magnification) showing time-dependent pattern of VEGF protein (TRITC-fluorescent) in capsaicin-treated Hy-A549 cells were represented along with nuclear staining (DAPI: blue). (E) Representative images of HUVEC migration upon incubation with (i) recombinant VEGF-supplemented control media, or VEGF-supplemented spent media of capsaicin-treated Hy-A549 cells, or with (ii) anti-VEGF-treated Hy-A549 spent media (*left panel*). Percent cell migrated in the wound area is being represented graphically (*right panel*). (F) Representative images of capillary-like sprout formation by HUVECs upon incubation with recombinant VEGF-supplemented spent media of capsaicin-treated Hy-A549 cells or with anti-VEGF-treated Hy-A549 spent media. GAPDH/α-Actin was used as internal loading control. Values are mean ±SEM of three independent experiments in each case or representative of typical experiment.

All the reactions so far defined occurred independent of direct contact of HUVEC cells with tumor cells or even proximity, thereby raising the possibility of the presence of tumor-shed soluble pro-migratory factors in the spent media. Supporting this, spent medium of Brefeldin-A-treated Hy-A549 failed to induce significant EC migration (Figure 2A) and capsaicin could not introduce any further effect (Figure 2A). To re-confirm our hypothesis, effect of capsaicin in regulating pro-angiogenic factors like VEGF, bFGF, EGF, TGF-β, was monitored in Brefeldin-A pre-treated Hy-A549 cells. Although capsaicin failed to alter bFGF, EGF and TGF-β levels it significantly down-regulated VEGF expression (Figure 2B). Further studies revealed that the spent media of Hy-A549 cells (10^6^ cells/ml of media) contained an average of 1.6 ng VEGF that decreased significantly after capsaicin treatment (Figure 2C, *left panel*). To adjudge the effect of capsaicin in regulating VEGF in Hy-A549 cells, we adopted few approaches. In the first approach, when Brefeldin A-pretreated Hy-A549 cells were treated with capsaicin for 24 h, intracellular VEGF was abrogated both at mRNA and protein levels (Figure 2C, *middle panel*). These observations were further supported by our quantitative real time PCR data depicting a decrease in relative VEGF mRNA expression in Hy-A549 cells following capsaicin treatment (Figure 2C, *right panel*) and re-confirmed by confocal microscopy (Figure 2D). In the second approach, control media supplemented with recombinant VEGF (0.8 ng/ml) increased endothelial cell migration and sprout formation (Figure 2E & 2F). In the third approach, when capsaicin-pretreated Hy-A549 spent media was supplemented with recombinant VEGF protein (0.8 ng/ml), reversal of capsaicin effect on EC migration and sprout formation was observed (Figure 2E & 2F). In the fourth approach, HUVECs showed significant reduction in migration (Figure 2E) and sprout formation (Figure 2F) when Hy-A549 cell spent medium was pre-treated with anti-VEGF antibody. However, anti-VEGF antibody failed to furnish any significant additional inhibitory effect on HUVEC migration and sprout formation in capsaicin pre-treated Hy-A549 spent media (Figure 2E & 2F). These results together validated the hypothesis that Hy-A549 cell-shed VEGF plays a crucial role in cancer-induced EC migration and sprout formation. Afore-furnished observations tempted us to demarcate the complete mechanism underlying the regulation of tumor angiogenesis by capsaicin.

### Capsaicin inhibits VEGF transcription by targeting HIF-1α

**Figure 3:**
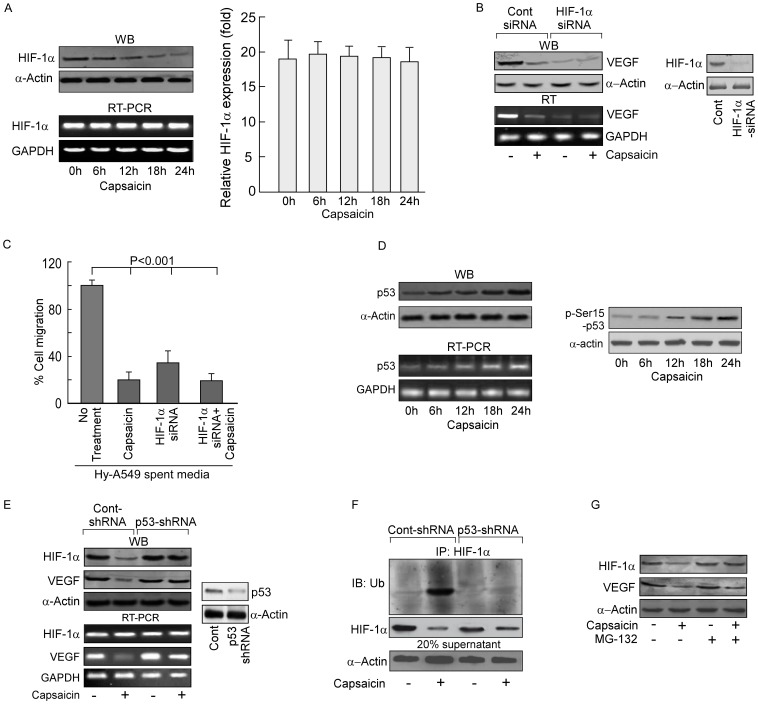
Capsaicin inhibits VEGF transcriptional activation by targeting HIF-1α in a p53-dependent manner. (A) Time-dependent expression profiles of HIF-1α mRNA and -protein were determined by Western blot and RT-PCR, respectively, in capsaicin-treated Hy-A549 cells (*left panel*). Capsaicin-treated Hy-A549 cells were examined for time-dependent variation in the expression profiles of HIF-1α by quantitative real time PCR analysis and represented graphically (*right panel*). (B) Hy-A549 cells, transiently transfected with a non-targeting control-siRNA or HIF-1α-siRNA, were incubated with/without capsaicin for 24 h; the cells were then analyzed to determine VEGF expression at protein and mRNA levels (*left panel*). Immunoblot showing transfection efficiency of HIF-1α (*right panel*) (C) Control-siRNA/HIF-1α-siRNA-transfected Hy-A549 cell-supernatants were used to assess HUVEC migration by wound healing assay after capsaicin-treatment (37.5 µM; 24 h) and represented graphically. (D) Time-dependent expression profile of p53-mRNA and -protein was determined by Western blotting and RT-PCR in capsaicin-treated Hy-A549 cells (*left panel*). p53 phosphorylation at Serine-15 position was also evaluated (*right panel*). (E) Hy-A549 cells, transfected with control-shRNA/p53-shRNA were incubated with capsaicin for 24 h and HIF-1α VEGF-mRNA and -protein were determined by Western blot and RT-PCR (*left panel*). Transfection efficiency was checked by analyzing p53 expression level (*right panel*). (F) HIF-1α was immunoprecipitated from capsaicin-treated Hy-A549 cell lysates and immunoblotted with anti-Ub antibody to assay HIF-1α ubiquitination. The ladder of bands represented ubiquitinated HIF-1α. In parallel experiment, immunoprecipitates were assayed for HIF-1α levels by Western blot. Comparable protein input was confirmed by direct Western blotting with anti-α-actin using 20% of the cell lysates that were used for immunoprecipitation. (G) Control and MG-132 drug-pretreated Hy-A549 cells were subjected to capsaicin-treatment for 24 h and then were examined for expression of HIF-1α/VEGF by Western blotting. α-Actin/GAPDH was used as internal loading control. Values are mean ±SEM of three independent experiments in each case or representative of typical experiment.

As capsaicin reduces VEGF both at the transcriptional as well as translational levels we hypothesized that this phytochemical might have a regulatory effect on HIF-1α, the main transcription factor of VEGF during hypoxia [4]. Interestingly, capsaicin reduced HIF-1α at protein level but not at mRNA level in Hy-A549 cells (Figure 3A, *left panel*), which was further confirmed by our quantitative real time PCR data (Figure 3A, *right panel*). Furthermore, HIF-1α-siRNA-transfected Hy-A549 cells furnished decrease in VEGF expression (Figure 3B) and spent media of these transfectants demonstrated significantly less EC migration (Figure 3C). These results indicated that capsaicin inhibited VEGF by down-modulating its key transcription factor HIF-1α (Figure 3C). However, since capsaicin treatment of these transfectants demonstrated additional decrease in VEGF, involvement of HIF-1α-independent pathway(s) of VEGF inhibition by capsaicin cannot be negated.

### Capsaicin targets HIF-1α in a p53-dependent manner

Since p53 directly targets HIF-1α for proteosomal degradation [9], we assessed the role of p53, if any, in capsaicin-induced regulation of HIF-1α and VEGF. Capsaicin treatment resulted in a time-dependent elevation of p53 in Hy-A549 cells (Figure 3D) though this augmentation in p53 level was much lower than that induced by the apoptotic dose (∼50 µM) of capsaicin [27], [28]. Additionally, capsaicin treatment increased the level of p-Ser15-p53 suggesting stability and functional activation of p53 (Figure 3D). Next, an increase in HIF-1α protein expression along with up-regulation in VEGF expression was observed in p53-shRNA-transfected Hy-A549 cells (Figure 3E), thereby indicating the possibility of involvement of p53 in capsaicin-induced anti-angiogenicity. Results of Figure 3F depicted significant HIF-1α ubiquitination in capsaicin-treated Hy-A549 cells while silencing p53 decreased the same (Figure 3F). Moreover, addition of the proteosome blocker MG-132 to Hy-A549 prior to capsaicin treatment partially increased the level of HIF-1α, although the effect was not comparable to that of p53-shRNA-transfected Hy-A549 cells (Figure 3G). These results tempted us to hypothesize that p53-dependent degradation of HIF-1α had a crucial role in capsaicin-mediated decrease in VEGF expression. Above results also indicate an additional role of p53 in maintaining transcriptional activity of HIF-1α Therefore, all these findings leave a room for exploring the status of the factors responsible for transporting HIF-1α to the nucleus, up on capsaicin treatment.

### Capsaicin inhibits the nuclear localization of HIF-1α by down regulating Cox-2 in a p53-dependent manner

**Figure 4:**
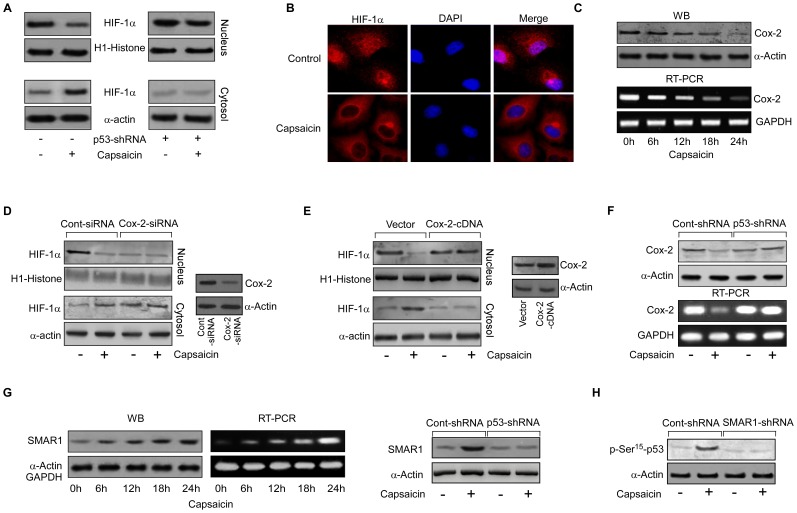
Capsaicin inhibits the nuclear localization of HIF-1α by down regulating Cox-2 in a p53-dependent manner. (A) Immunoblot representing nuclear and cytosolic levels of HIF-1α in capsaicin-treated/p53-shRNA-transfected Hy-A549 cells. (B) HIF-1α expression was monitored in capsaicin-treated Hy-A549 cells by confocal microscopy (magnification 60x). (C) Time-dependent expression of Cox-2-mRNA and -protein in capsaicin-treated Hy-A549 cells was determined by Western blot and RT-PCR. (D) Control siRNA-/Cox-2-siRNA-transfected Hy-A549 cells were treated with capsaicin (37.5 µM; 24 h) and nuclear translocation of HIF-1α was assessed by Western blot analysis (*left panel*). Transfection efficiency was determined by analyzing the expression of Cox-2 (*right panel*). (E) Control vector-/Cox-2 cDNA transfected Hy-A549 cells were treated with capsaicin (37.5 µM; 24 h) and analyzed for nuclear and cytosolic expression of HIF-1α (*left panel*). Transfection efficiency of Cox-2 was also verified (*right panel*). (F) Control-shRNA-/p53-shRNA-transfected Hy-A549 cells were treated with capsaicin (37.5 µM; 24 h) and expression profiles of Cox-2 both at protein and mRNA level were examined. (G) Time-dependent expression profiles of SMAR1-protein and mRNA in capsaicin-treated Hy-A549 cells were determined by Western blot and RT-PCR (*left panels*). Control-/p53-shRNA transfected Hy-A549 cells were treated with capsaicin and expression levels of SMAR1 were checked (*right panel*). (H) Control-/SMAR1-shRNA transfected Hy-A549 cells were treated with capsaicin and immune-blotted with p-Ser^15^-p53. α-Actin/H1-Histone/GAPDH were used as internal loading control. Values are mean ± SEM of three independent experiments in each case or representative of typical experiment.

To understand the role of p53, if any, in controlling nuclear translocation of the transcription factor HIF-1α, the nucleus to cytoplasmic ratio of HIF-1α was checked up on capsaicin-treatment. Results of Figure 4A demonstrated a sharp decrease in nuclear HIF-1α level with its increase in cytoplasm, indicating the inhibition in the transcriptional activity of HIF-1α in capsaicin-treated Hy-A549 cells. Confocal microscopic data authenticated these results (Figure 4B). Silencing p53 reversed this effect (Figure 4A) indicating that p53 is responsible also for obstructing HIF-1α transport to the nucleus up on capsaicin treatment. It is well acknowledged that intra-cellular PGE2, which is synthesized by the pro-inflammatory enzyme Cox-2 [5], is a determinant of nuclear localization of HIF-1α [29]. Interestingly, capsaicin-treatment inhibited Cox-2 expression both at the transcriptional and protein levels (Figure 4C). Consistently, while silencing Cox-2 abrogated the nuclear localization of HIF-1α (Figure 4D), over-expression of Cox-2 increased the same in Hy-A549 cells (Figure 4E). Moreover, knocking-down of wild-type p53 elevated Cox-2 expression both at mRNA and protein levels (Figure 4F) in Hy-A549 cells. These results showing inverse regulation of Cox-2 by p53 raised the question as to whether p53 mediates Cox-2 down-regulation directly or through some other p53-regulated molecule(s).

### Capsaicin encourages functioning of p53-SMAR1 positive feed-back loop

Being a multifunctional protein, p53 forms molecular complexes with different DNA targets and interacts with a number of cellular proteins, e.g., Mdm2, Gadd45, p21, Bax, 14-3-3, SMAR1, Apaf-1 etc., of which SMAR1 has been well-documented as a transcriptional repressor [30]. Interestingly, while capsaicin treatment led to a time-dependent increase in SMAR1 both at protein as well as mRNA levels (Figure 4G, *left panel*) in Hy-A549 cells, it failed to do the same in cells stably transfected with p53-shRNA (Figure 4G, *right panel*) emphasizing that capsaicin induces SMAR1 expression in p53-dependent manner. Interestingly, SMAR1-shRNA transfection significantly decreased phospho-p53 level in the transfectants thereby hindering p53 activation, which capsaicin failed to affect further (Figure 4H). These results, therefore, suggest the existence of positive interdependence between p53 and SMAR1 where p53 activates SMAR1 transcription and SMAR1 in turn stabilizes p53 to facilitate its function [31].

### Capsaicin activates SMAR1 to downregulate Cox-2 transcription

**Figure 5:**
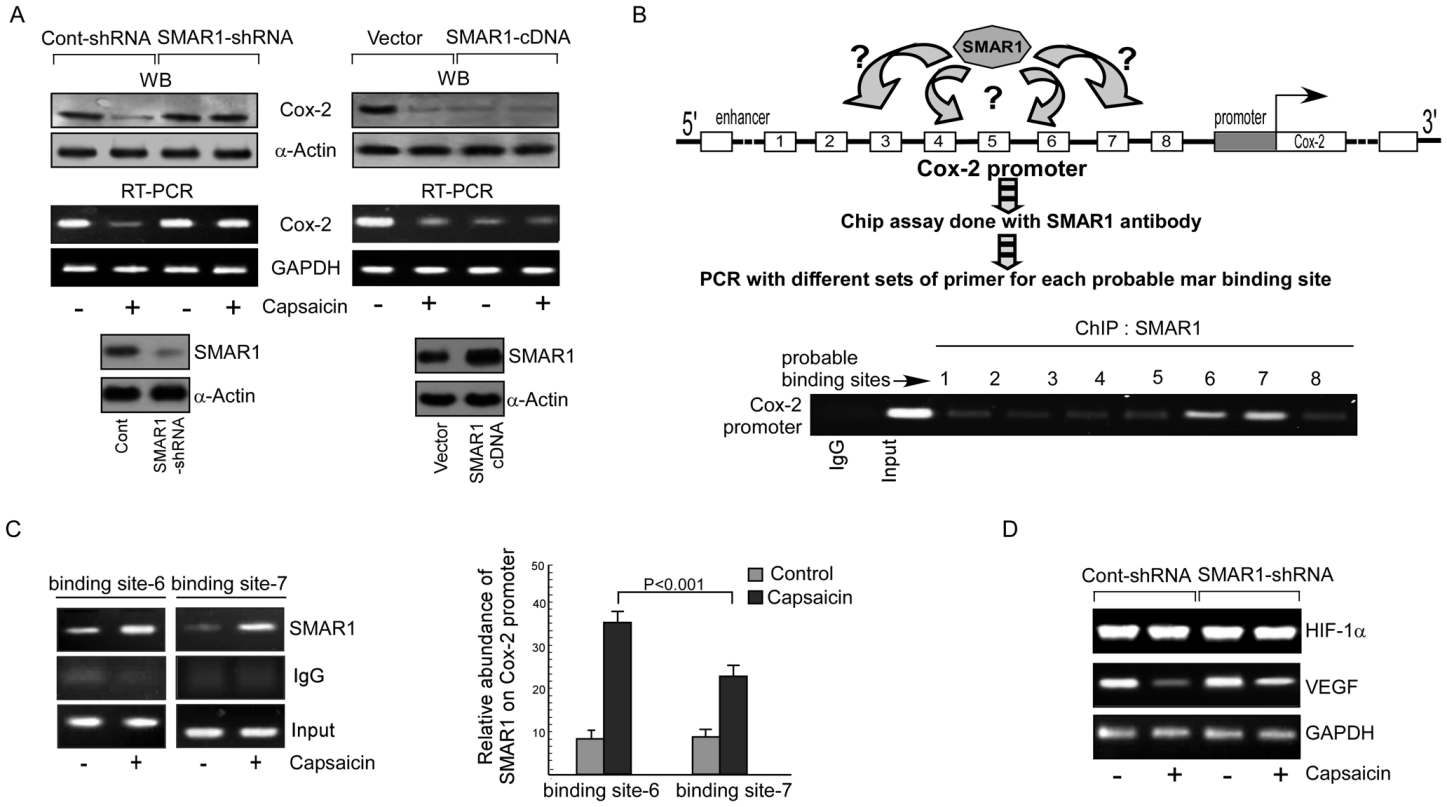
Capsaicin suppresses VEGF expression by SMAR1-mediated down regulation of Cox-2. (A) Control-/SMAR1-shRNA (*left panel*) or control-vector/SMAR1-cDNA (*right panel*) transfected Hy-A549 cells were treated with capsaicin were assayed for Cox-2 expression by Western blot and RT-PCR. Transfection efficiency was verified by Western blot (*bottom panels*). (B) Cox-2 promoter activity was checked in Hy-A549. Schematic representation of the Cox-2 promoter showing the probable SMAR1-binding sites predicted by using the MARWIZ software, PCR run with eight different sets of primers designed for each probable MAR binding site spreading over four regions (−141 bp to −421 bp; −631 bp to −1121 bp; −1262 bp to −1331 bp; −1471 bp to −1891 bp). ChIP assay with anti-SMAR1 was performed on MAR-binding regions of Cox-2 promoter. Input and control IgG was used as internal control and negative control. (C) The relative abundance of SMAR1 on Cox-2 promoter was analyzed in control and capsaicin treated Hy-A549 cells at binding sites 6 & 7 after ChIP of Cox-2 promoter with anti-SMAR1(*left panel*) and represented graphically (*right panel*). (D) Control-/SMAR1-shRNA-transfected Hy-A549 cells were treated with capsaicin and analyzed for reporter HIF-1α and VEGF gene expression by RT-PCR. α-Actin/GAPDH were used as internal loading control. Values are mean ±SEM of three independent experiments in each case or representative of typical experiment.

Next to assess the role of SMAR1, if any, in Cox-2 expression, Hy-A549 cells were transfected with SMAR1-shRNA or SMAR1-cDNA prior to capsaicin treatment. While SMAR1-shRNA transfection reverted back the effect of capsaicin on Cox2 expression both at transcriptional as well as translational level (Figure 5A, left panel), SMAR1 over-expression significantly repressed Cox-2 expression (Figure 5A, right panel). Capsaicin treatment failed to add any further effect in these transfectants thereby suggesting not only that SMAR1 might be acting as the transcriptional repressor of Cox-2 but also that capsaicin inhibits Cox-2 expression *via* SMAR1. By bioinformatics analysis using MARWIZ software analyzer, Cox-2 promoter sequence (141 bp upstream of the transcription start site) was screened for the presence of MAR-binding sites. Our analyses predicted eight probable binding sites for SMAR1 on Cox-2 promoter (Figure 5B). To further confirm that SMAR1 is directly recruited to the Cox-2 promoter in Hy-A549 cells, different sets of overlapping primers of the predicted MAR-binding sites were designed for a DNA chromatin immunoprecipitation (ChIP) experiment to locate the binding site of SMAR1 on Cox-2 promoter. Figure 5B depicted the recruitment of SMAR1 in the −1471 to −1891 bp region (binding sites 6 and 7) upstream of the Cox-2 gene in Hy-A549 cells which was further strengthened after capsaicin treatment (Figure 5C). Consistently, stable transfection of SMAR1-shRNA in Hy-A549 cells directed to augmentation in HIF-1α and VEGF (Figure 5D). These results not only corroborate the direct recruitment of SMAR1 on Cox-2 promoter sequence but also identify SMAR1 as the molecule directly responsible for capsaicin-induced repression of Cox-2 expression in NSCLC under hypoxic condition as a consequence of which VEGF expression was down-regulated.

In a nutshell, capsaicin-induced VEGF down-regulation in NSCLC cells under hypoxic condition is mediated by re-activation of p53-SMAR1 auto-regulatory loop that ensures down-regulation of HIF-1α, the major transcription factor of VEGF. Such signal modulation consequently blocks lung cancer-induced EC migration and tube formation, pre-requisites of tumor angiogenesis.

## Discussion

Highly resistant non-small cell lung cancer is one of the major causes of cancer death across the world and angiogenesis has emerged as an integral process in promoting the growth and metastasis of NSCLCs. Inhibition of VEGF, one of the key mediators of angiogenesis, by molecular-targeting agent may, therefore, be an important approach towards development of potential anticancer therapy for regressing NSCLC. However, in spite of modest positive outcome with the use of anti-angiogenic drugs based on some clinical trials [32], [33], no long-term survival benefits have been documented as yet for different cancers [34]. In addition, toxicity of most of these drugs towards normal cells as well as development of drug-resistance in tumor cells necessitated investigations into alternative compounds to improve current therapeutic management. Recently capsaicin has been recognized for its pharmacological and toxicological properties and for selectively suppressing the growth of various human tumor cell lines [13]–[18]. However, although there is considerable clinical interest in regressing NSCLC by halting tumor-angiogenesis [35], there is no scientific evaluation of the molecular mechanisms underneath the effect of capsaicin in the management of NSCLC-induced angiogenesis. The present study portrayed detailed molecular mechanisms underlying the anti-angiogenic effect of capsaicin, the major pungent ingredient from red chili pepper.

According to Patel *et al*. [36], capsaicin treatment inhibited NF-κB activation and cell proliferation, but enhanced VEGF production by enhancing HIF-1α expression and binding to hypoxia response element (HRE) in human malignant melanoma cells. These findings support the hypothesis that inhibition of growth-signaling pathways by capsaicin might trigger tumor cells to produce paracrine factors such as VEGF, critical for neovascularization, allowing tumors to survive and progress. Contradicting this hypothesis, Min *et al*. [23] demonstrated suppression of human fibrocarcoma-induced angiogenesis in chick chorioallantoic membrane assay by capsaicin. They further demonstrated that capsaicin directly inhibited VEGF-induced proliferation, DNA synthesis, chemotactic motility, and capillary-like tube formation of human endothelial cells [23]. However, that report did not include the effect of capsaicin on VEGF production by the tumor cells. Although Patel *et al*. [37] demonstrated that capsaicin enhanced production of VEGF by melanoma cells we did not observe similar induction of VEGF following capsaicin treatment. Our results demonstrated that capsaicin acted directly on tumor angiogenesis by suppressing VEGF expression in hypoxic NSCLC cells while having minimal toxic effects on normal cells. This difference might be due to differences in the cell lines used in these studies. In fact, the anti-proliferative and anti-tumor effects of capsaicin on *in vivo* lung cancer models [38] failed to support the role of capsaicin as a VEGF inducer for neo-vascularization that allows lung cancer to survive and progress. Our work established the mechanism of capsaicin-induced down-regulation in VEGF expression in our NSCLC model. It is well acknowledged that hypoxia-conducive environment, resulting from the increasing distance between the growing tumor cells and the capillaries or from the inefficiency of new vessels, resulted in rapid up-regulation of VEGF [39] and its receptor *via* HIF-1α [40]. We observed that in hypoxic NSCLC cells, capsaicin re-activated the auto-regulatory p53-SMAR1 signaling loop that in turn ensured down-regulation of HIF-1α, the major transcription factor of VEGF. Effect of such signal modulation consequently blocked tumor-induced endothelial cell migration and tube formation, pre-requisites of tumor angiogenesis.

Scaffold/matrix attachment regions (S/MARs) are regulatory DNA sequences mostly present upstream of various promoters. Matrix attachment region-binding proteins (MARBPs), which bind to such regulatory sequences, interact with numerous chromatin modifying factors and facilitate transcription in response to diverse stimuli [41]. SMAR1 is an MARBP identified in mouse double positive thymocytes, wherein it binds to MARβ sequence at TCRβ locus and affects V(D)J recombination [42], [43]. Subsequently, SMAR1 has been characterized as a tumor suppressor by virtue of its ability to interact with p53 and delay tumor growth in mouse melanoma model [38], [39]. In fact, p53 target gene SMAR1 [44] activates and stabilizes p53 [8] with which it acts synergistically during DNA damage [44]. Coordinated regulation of p53 apoptotic targets by SMAR1 has also been documented [10]. However, the biological and functional significances of p53 and SMAR1 cross-talk in the context to VEGF expression by non-small cell lung cancer are not known.

**Figure 6:**
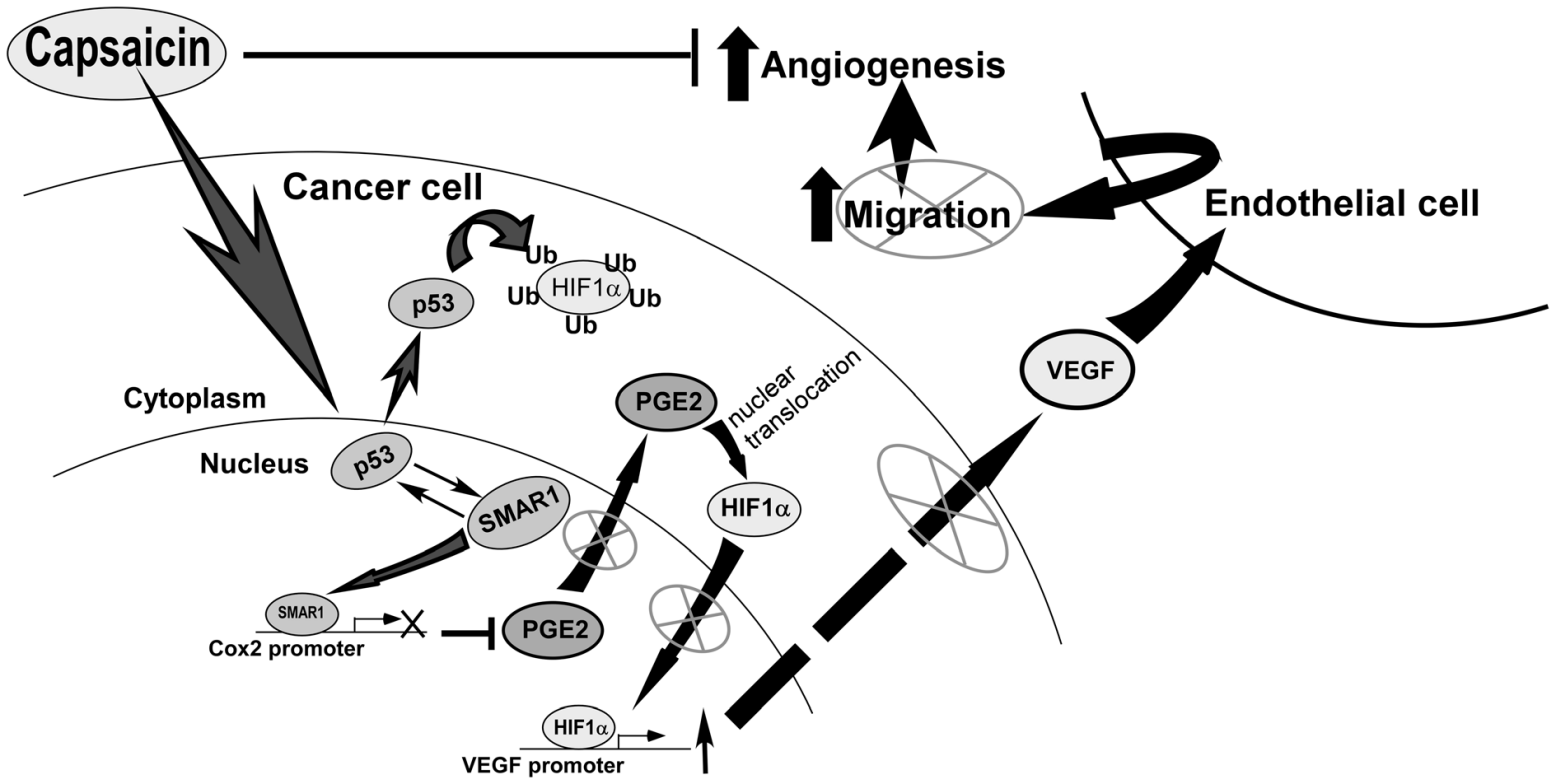
Schematic diagram representing molecular mechanisms of capsaicin-mediated down-regulation of pro-angiogenic factor, VEGF.

In this study, we showed that capsaicin reactivated p53-SMAR1 auto-regulatory signaling loop in NSCLC cells where p53 transactivated SMAR1 that in turn stabilized p53. Activated p53 then down-regulated HIF-1α by (i) facilitating degradation of HIF-1α and (ii) inhibiting its transcription. In addition, p53-SMAR1 cross-talk negated the possibility of the remaining HIF-1α to traverse to the nucleus for VEGF trans-activation, by repressing the expression COX-2, the enzyme synthesizing PGE2 [5] which is a determinant of nuclear localization of HIF-1α [29]. All these molecular cross-talks dampened VEGF expression in NSCLC cells to finally inhibit NSCLC-induced endothelial cell network formation, pre-requisite of angiogenesis (Figure 6). To our knowledge, this is the first report establishing that SMAR1 acts in collaboration with p53, as a repressor of VEGF expression to finally restrict non small-cell carcinoma-induced angiogenesis. Thus, the molecular regulation of p53-SMAR1 positive feed-back loop may be a potential therapeutic strategy for non small-cell lung carcinoma.

In summary, as a novel strategy to maximize the effectiveness of targeted therapies and to minimize the impact of side effects of available cytotoxic drugs, we have identified the efficacy of capsaicin in targeting p53-SMAR1 auto-regulatory loop to inhibit resistant non small-cell lung carcinoma-induced angiogenesis.
